# Hepatitis C-Associated Mixed Cryoglobulinemic Vasculitis Induces Differential Gene Expression in Peripheral Mononuclear Cells

**DOI:** 10.3389/fimmu.2014.00248

**Published:** 2014-05-27

**Authors:** Sreetha Sidharthan, Cheol-Woo Kim, Alison A. Murphy, Xiaozhen Zhang, Jun Yang, Richard A. Lempicki, Michael C. Sneller, Shyam Kottilil

**Affiliations:** ^1^Critical Care Medicine Department, Clinical Center, National Institutes of Health, Department of Health and Human Services, Bethesda, MD, USA; ^2^Department of Internal Medicine, Inha University, Incheon, South Korea; ^3^Laboratory of Immunoregulation, National Institute of Allergy and Infectious Diseases, National Institutes of Health, Department of Health and Human Services, Bethesda, MD, USA; ^4^Clinical Research Directorate/Clinical Monitoring Research Program, Leidos Biomedical Research Inc., Frederick National Laboratory for Cancer Research, Frederick, MD, USA

**Keywords:** hepatitis C, mixed cryoglobulinemia, vasculitis, interferon-stimulated genes

## Abstract

This study examines the distinct gene expression profile of peripheral blood mononuclear cells from patients with chronic hepatitis C infection and mixed cryoglobulinemic (MC) vasculitis. Our DNA microarray analysis indicates that hepatitis C virus (HCV)-associated MC vasculitis is characterized by compromised neutrophil function, impaired chemotaxis, and increased interferon-stimulated gene (ISG) expression, contributing to overall MC pathogenesis and end-organ damage. Increased ISG expression is suggestive of an enhanced endogenous interferon gene signature. PBMC depletion assays demonstrate that this increased expression is likely due to an activation of monocytes and not a direct result of B cell expansion. Notably, this monocyte activation of ISG expression in HCV-associated MC vasculitis suggests a poor predictor status of interferon-based treatment. Further analysis of PBMC gene expression profiles before and after *in vivo* B cell depletion therapy is critical to completely understanding the mechanisms of MC vasculitis pathogenesis.

## Introduction

Chronic hepatitis C (CHC) infection affects 180 million people worldwide (1). Hepatitis C virus (HCV) is a single-stranded RNA virus that preferentially infects hepatocytes of the liver (2). CHC can lead to progressive liver fibrosis, cirrhosis, hepatocellular carcinoma, and death (3). CHC has also been associated with several extrahepatic manifestations, among which include mixed cryoglobulinemic (MC) vasculitis, lymphoproliferative disorders, and insulin resistance (4, 5). Of these, Type II MC vasculitis is the most common and directly attributed to CHC (6), with CHC identified in more than 80% of patients with persistent MC vasculitis (7, 8).

Hepatitis C virus-associated MC vasculitis is characterized by an expansion of monoclonal B cells probably triggered by HCV antigens (6, 9). These clonal B cells produce IgM with rheumatoid factor activity, which can lead to the formation and deposition of immune complexes and eventual small vessel vasculitis (6, 9). The end result is tissue and organ damage, particularly of the kidneys and skin, the common clinical manifestations being membranoproliferative glomerulonephritis and cutaneous vasculitis (4, 10).

Recent studies have demonstrated that patients diagnosed with MC vasculitis can be effectively treated with B cell depletion therapy (11–13). However, the role of B cells in a variety of immunological abnormalities associated with MC and the exact nature of how CHC infection affects the pathogenesis of MC vasculitis are not completely understood. In particular, whether monoclonal B cell expansion can lead to activation of other immune cells to further contribute to the pathogenesis is not clear. In this study, we performed gene expression profile analysis of PBMCs from four different patient cohorts to determine the change in gene expression among HCV-associated MC vasculitis patients compared to other subjects with and without HCV and vasculitis.

## Materials and Methods

### Study design and patient population

PBMCs were isolated by venipuncture from normal volunteers (*N* = 12), HCV monoinfected subjects (*N* = 7), human immunodeficiency virus (HIV) and HCV coinfected subjects on antiretroviral therapy (*N* = 5), and HCV MC vasculitis subjects (*N* = 7) (Table 1). HIV was diagnosed by ELISA and Western blot using the Cambridge Biotech HIV-1 Serum Western Blot Kit (Maxim Biomedical, Rockville, MD, USA). HIV RNA was quantified by Versant HIV RNA 3.0 Assay (Bayer Diagnostics, Puteaux Cedex, France). HCV genotype was determined by the INNO-LiPA HCV II (Innogenetics) assay and HCV RNA was quantified by the Versant HCV RNA 3.0 Assay (Bayer Diagnostics, Tarrytown, NY, USA).

**Table 1:** **Demographics and clinical characteristics of study participants**.

Group	Age	Gender	Race	Risk	HCV GT	HCV VL	HIV Ab	HIV VL	TCD4	CD4%	Systemic corticosteroids	Clinical manifestations of MC vasculitis
Normal volunteer 1	41	F	White	N/A	N/A	N/A	Negative	N/A				
Normal volunteer 2	34	M	White	N/A	N/A	N/A	Negative	N/A				
Normal volunteer 3	56	F	White	N/A	N/A	N/A	Negative	N/A				
Normal volunteer 4	37	F	Black	N/A	N/A	N/A	Negative	N/A				
Normal volunteer 5	42	M	White	N/A	N/A	N/A	Negative	N/A				
Normal volunteer 6	46	F	White	N/A	N/A	N/A	Negative	N/A				
Normal volunteer 7	39	M	Hispanic	N/A	N/A	N/A	Negative	N/A				
Normal volunteer 8	45	M	White	N/A	N/A	N/A	Negative	N/A				
Normal volunteer 9	38	F	White	N/A	N/A	N/A	Negative	N/A				
Normal volunteer 10	46	M	White	N/A	N/A	N/A	Negative	N/A				
Normal volunteer 11	41	F	Black	N/A	N/A	N/A	Negative	N/A				
Normal volunteer 12	27	W	Male	N/A	N/A	N/A	Negative	N/A				
HIV/HCV 1	49	M	Black	IVDU	1a	7,692,310	Positive	121	1,233	45		
HIV/HCV 2	40	M	Black	MSM	1a	4,976,400	Positive	<50	1,460	44		
HIV/HCV 3	51	M	Black	IVDU	1b	3,945,420	Positive	<50	727	30		
HIV/HCV 4	49	F	Black	IVDU	1a	1,054,510	Positive	<50	794	25		
HIV/HCV 5	55	M	Black	MSM	1b	9,504,730	Positive	<50	1,008	43		
HCV 1	51	M	White	IVDU	1b	2,500,000	Negative	N/A				
HCV 2	53	F	White	IVDU	1a	473,000	Negative	N/A				
HCV 3	51	M	Black	IVDU	1	441,000	Negative	N/A				
HCV 4	45	M	White	IVDU	1a	3,820,000	Negative	N/A				
HCV 5	42	M	White	IVDU	2	10,900,000	Negative	N/A				
HCV 6	59	F	Black	IVDU	2b	7,810,000	Negative	N/A				
HCV 7	70	M	White	IVDU	1b	3,830,000	Negative	N/A				
HCV MC Vasc 1	56	M	White	Needlestick	1a	3,419,770	Negative	N/A				Arthralgia, purpura, peripheral neuropathy
HCV MC Vasc 2	52	M	White	Transfusion acquired	1a, 1b	1,864,910	Negative	N/A			Prednisone 10 mg daily	Arthritis, purpura, peripheral neuropathy
HCV MC Vasc 3	47	F	White	IVDU	2b	2,296,250	Negative	N/A				Peripheral neuropathy, hematuria
HCV MC Vasc 4	56	M	White	Intranasal cocaine	1a	50,816	Negative	N/A				Purpura, glomerulonephritis
HCV MC Vasc 5	47	M	White	Intranasal cocaine	1a	932,880	Negative	N/A			Prednisone 50 mg daily	Purpura, ulcers, mononeuritis
HCV MC Vasc 6	56	M	White	IVDU	1a	100,907	Negative	N/A				Arthralgia, purpura, peripheral neuropathy
HCV MC Vasc 7	58	F	White	IVDU	1	<615	Negative	N/A			Prednisone 30 mg daily	Purpura, ulcers, hematuria

*HIV and HCV viral loads were measured by the Versant RNA 3.0 Assay with a lower limit of detection of 50 copies/ml and 615 IU/ml, respectively. qRT-PCR results are shown only for 5 of the 12 healthy volunteers and 6 of the 7 HCV monoinfected subjects due to availability of samples. Samples for the other seven normal volunteer patients were used to validate microarray analysis in Kottilil et al. (14). Additionally, B cell depletion experiments were only done on subsets of individuals as determined by sample availability*.

Normal volunteers were selected through the blood bank and were HIV and HCV negative. HIV/HCV coinfected patients and the HCV monoinfected patients were selected from an ongoing longitudinal study at the National Institutes of Health (NIH). The HCV MC vasculitis subjects were selected from an ongoing open-label, randomized controlled trial conducted at the NIH (13). Inclusion in this trail required the presence of active manifestations of MC vasculitis as described in Sneller et al. (13). Only patients who did not respond to or tolerate interferon-alpha and ribavirin over a year before start of this study were enrolled. Patients were allowed to continue taking corticosteroids, but samples were collected before the day’s dose, at steroid trough level, to minimize the immunomodulatory effects of these drugs. All donors signed informed consents approved by the National Institute of Allergy and Infectious Diseases Institutional Review Board. Clinical protocols NCT00029107, NCT00001281, and NCT00076427 were used to enroll study subjects.

### Isolation of PBMCs and RNA

PBMCs were isolated from white blood cells by the standard Ficoll-Hypaque Plus (Amersham Biosciences, Uppsala, Sweden) density gradient separation technique and then frozen for storage. RNA was isolated using the Qiagen mRNA isolation kit (Qiagen, Germantown, MD, USA) following the manufacturer’s protocol and to be used for DNA microarray and quantitative reverse transcription polymerase chain reaction (qRT-PCR) analysis.

### DNA microarray analysis

Complementary RNA was prepared from total RNA and hybridized to Affymetrix U133A 2.0 oligonucleotide arrays according to the manufacturer’s protocols (Affymetrix, Santa Clara, CA, USA) as previously described (14). A significant analysis of microarray (SAM) algorithm was used to determine the genes that were differentially expressed after an extensive filtering process. Genes with low variability or undetectable expression levels (for the majority of samples) were eliminated from analysis if the Guanosine–Cytosine Robust Multi Array values for these genes were within the interquartile range of <0.263 or a 75th percentile of <5. The corresponding genes and samples from the individuals were then subjected to hierarchical clustering.

### Real-time quantitative reverse transcription polymerase chain reaction

Total RNA isolated from PBMCs was reverse-transcribed using random primers with the High Capacity cDNA Reverse Transcriptase Kit (Life Technologies). Between 1 and 25 ng of RNA was used for each quantitative qRT-PCR reaction. Taqman expression assays were run with technical duplicates except where indicated (Life Technologies). Primer/probe sets were pre-designed for respective genes and purchased from Life Technologies. Gene expression was determined as a cycle at threshold (Ct) based on 40 PCR cycles. For statistical analysis, undetectable expression was assigned a minimal detectable level with a Ct value of 40. Expression of *GAPDH* was used as an endogenous control, with *GAPDH* Ct values for all samples being distributed between 20 and 25. Relative expression of targets was calculated as dCt values (normalized by *GAPDH* Ct values) or ddCt values (to calculate fold change compared to other samples). Expression reactions were run in 96-well plates on a 7500 Real-Time PCR System (Applied Biosystems).

### Statistical analysis

ANOVA with Tukey’s multiple comparison test was used to compare means of the relative gene expression in independent groups. The paired *T*-test with the Bonferroni adjustment for multiple testing was used to compare paired responses.

### PBMC depletion and enrichment assay

Total PBMCs were isolated using Ficoll-Hypaque density gradient centrifugation. B cells (CD19 pan B dynabeads) and monocytes (CD14 dynabeads) were either depleted or enriched using an antibody-coated magnetic bead column separation technique (Life Technologies, Grand Island, NY, USA). Dynabeads CD19 pan B and CD14 are both uniform (4.5 μm diameter) superparamagnetic beads coated with a primary monoclonal antibody specific for the CD19 or CD14 membrane antigen mainly expressed on human B cells and monocytes, respectively. Cell purity for B cells was performed as follows using flow cytometry and was determined to be >90%. Cells were stained for CD3 APC (Clone UCHT1) and CD20 FITC (Clone: L27) (source for both antibodies: BD Biosciences, San Jose, CA, USA) and analyzed using a FACSCanto Cell Analyzer (BD Biosciences, San Jose, CA, USA). Cells were analyzed using a lymphocyte gate and purity was assessed as CD3^−^CD20^+^ lymphocytes. For monocytes, purity was assessed by staining cells for CD3 APC (Clone UCHT1) and intracellular CD68 FITC (Clone Y1/82A) (source: BD Biosciences, San Jose, CA, USA) after permeabilization and fixing. The cells were analyzed using a monocyte gate and purity assessed as CD3^−^CD68^+^ cells.

## Results

### Study subjects

Five patient cohorts were analyzed: normal volunteers, HCV monoinfected subjects, HIV/HCV coinfected subjects, and HCV MC vasculitis subjects (Table 1).

Human immunodeficiency virus/hepatitis C virus coinfected patients were of interest to us because coinfection with HCV is present in one-third of all HIV-infected individuals and the gene expression profiles of coinfected patients has already been characterized (14). Additionally, HIV infection drives T cell activation and MC vasculitis drives B cell activation. This patient group thus serves as an additional comparison to better understand how the pathogenesis of HCV-associated MC vasculitis pathogenesis induces a gene expression profile distinct from the other common HCV-infected patient cohorts.

Hepatitis C virus MC vasculitis subjects were selected based on the presence of active manifestations of CHC-associated MC vasculitis and required the presence of peripheral neuropathy, cutaneous vasculitis, and/or glomerulonephritis. Common clinical manifestations included purpura, arthralgia, hematuria, and ulcers. Patients with the presence of potentially life-threatening vasculitis involving the heart, central nervous system, or gastrointestinal tract were excluded. It should also be noted that three out of the seven HCV MC vasculitis patients were on prednisone and did not have a change in immunosuppressive therapy within 4 weeks of study entry. For these study subjects, samples were drawn as trough (23 h post morning dose of prednisone) to minimize the effect of corticosteroids on the immune system. Additionally, HCV MC vasculitis patients with prior use of rituximab, severe renal insufficiency, severe hepatic insufficiency, lymphoma, coinfection with HIV or hepatitis B virus, liver transplantation, or active systemic infections were excluded (13).

### Differential gene expression profiles in PBMCs of HCV MC vasculitis subjects

To compare the host gene expression profile induced by HCV infection with MC vasculitis to that induced by HCV monoinfection and HCV/HIV coinfection without MC, we performed DNA microarray analysis using RNA isolated from PBMCs from the aforementioned patient groups (Table 1).

**Figure 1:**
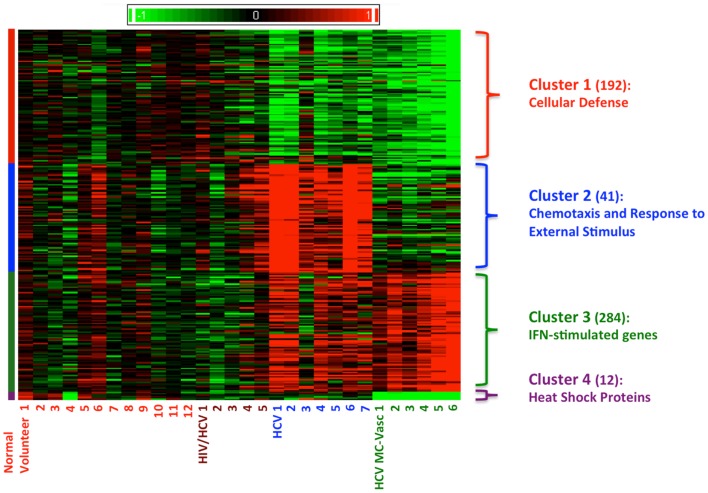
**Clustering of differentially expression genes in PBMCs from four patient cohorts**. Levels of gene expression were assayed using Affymetrix Human Genome U133A chips as described in the methods section. A total of 529 differentially expressed genes were identified. Genes were grouped using *K*-means clustering, and samples were grouped by hierarchical clustering. Differences in relative levels of gene expression (*Z*-score) are represented by color, where red indicates up-regulation and green indicates down-regulation relative to that of corresponding gene expression in controls. The numbers in parentheses specify the number of genes in each cluster. Cluster 1 consists of genes down-regulated in HCV monoinfected patients and HCV MC vasculitis patients. Cluster 2 consists of genes up-regulated in HCV monoinfected patients without vasculitis. Cluster 3 consists of genes up-regulated in HCV MC vasculitis patients and Cluster 4 consists of genes down-regulated in HCV MC vasculitis patients.

Using Affymetrix human genome U133A oligonucleotide arrays and a SAM algorithm, we identified a total of 529 differentially expressed genes between the four groups (Figure 1). The corresponding genes and samples from the individuals were subjected to hierarchical clustering, which revealed four distinct clusters of differential gene expression (Figure 1). Cluster 1 consists of 192 genes that are down-regulated in both HCV monoinfected patients and HCV MC vasculitis patients. Functional annotation analysis revealed that these genes share roles in cellular defense. Cluster 2 includes 41 genes that are up-regulated in HCV monoinfected patients when compared to HCV MC vasculitis subjects and are mostly genes implicated in chemotaxis and response to external stimulus. Cluster 3 consists of 284 genes including several interferon-stimulated genes (ISGs) that are up-regulated only in HCV MC vasculitis patients. Cluster 4 includes 12 heat shock proteins that are down-regulated in HCV MC vasculitis patients.

Representative genes that belong to each cluster were identified by rigorous literature-mining algorithms, significance of microarray analysis, and biology of the disease processes of both HCV viremia and MC vasculitis (Tables A1–A3 in Appendix) and validated by qRT-PCR. This process of gene selection is consistent with our previous studies (14).

### Down-regulation of cellular defense genes in HCV monoinfected patients with or without MC vasculitis

To validate our DNA microarray analysis, we performed qRT-PCR analysis on the most biologically relevant genes from each cluster selected as described above. Total RNA was extracted from the PBMCs and subjected to qRT-PCR using primers specific for the validated genes.

**Figure 2:**
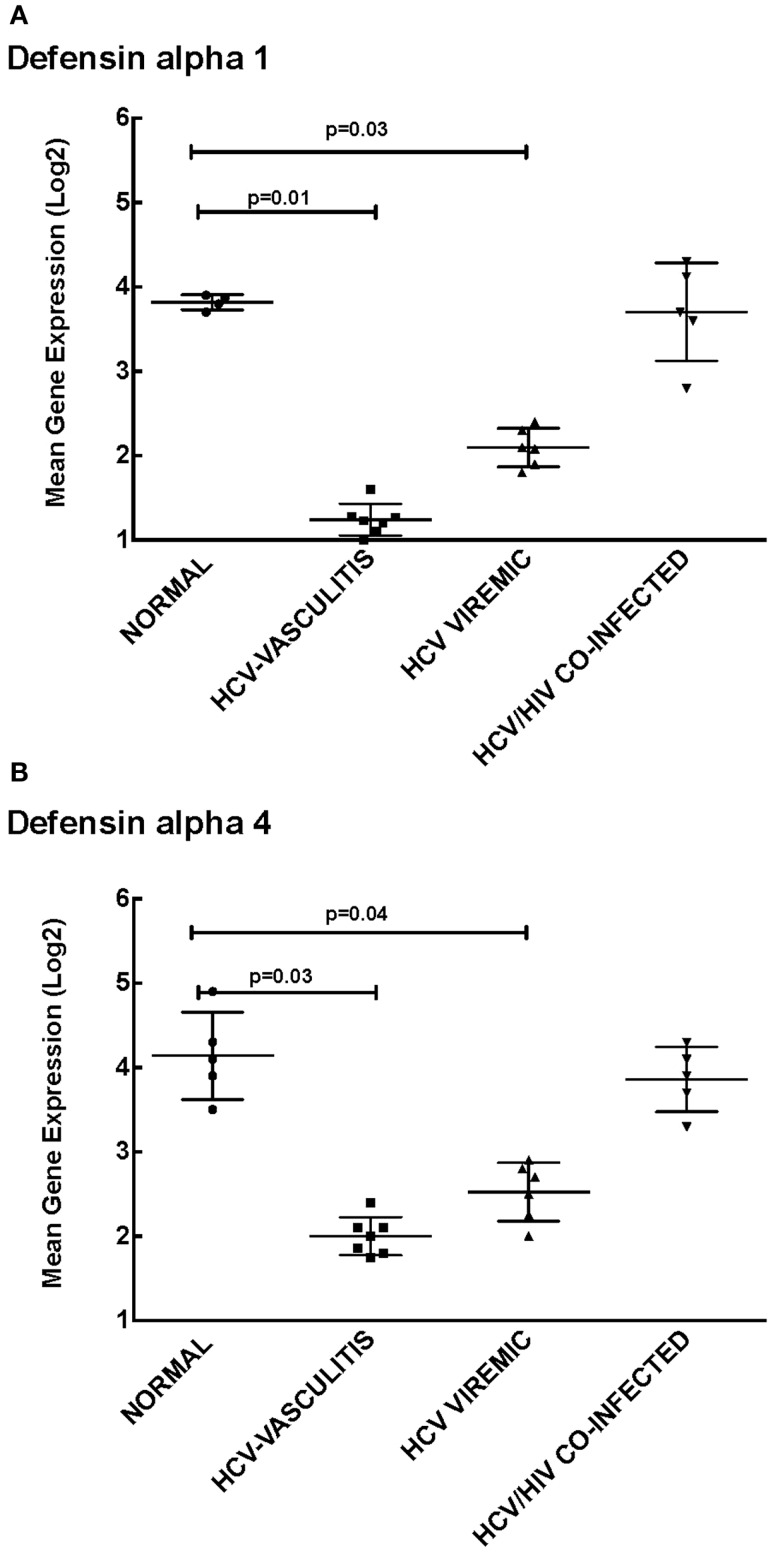
**Validation of Cluster 1 microarray data by qPCR analysis**. DNA microarray expression of biologically relevant genes selected from Cluster 1 was validated using qRT-PCR as described in the Section “Materials and Methods.” Levels of mean gene expression were calculated in comparison to the expression of *GAPDH*. The expression of defensin alpha-1 **(A)** and defensin alpha-4 **(B)** were lower in the HCV monoinfected (*p* = 0.03 and 0.04 with normal, respectively) and HCV MC vasculitis cohorts (*p* = 0.01 and 0.03 with normal, respectively).

Cluster 1 was comprised of genes involved in cellular defense, which were down-regulated in patients monoinfected with HCV, with or without vasculitis. We chose to measure transcript levels of defensin alpha-1 and defensin alpha-4 (Figure 2). The mean gene expression of defensin alpha-1 was significantly lower in HCV monoinfected and HCV MC vasculitis patients compared to the other two groups [3.80 ± 0.80 (NV), 1.20 ± 0.40 (HCV MC-Vasc), 2.10 ± 0.60 (HCV), 3.7 ± 0.70 (HIV/HCV); *p* = 0.01 between HCV MC-Vasc and normal and *p* = 0.03 between HCV viremic and normal], thereby confirming the DNA microarray analysis. This was similarly true for defensin alpha-4 [4.30 ± 0.40 (NV), 2.00 ± 0.90 (HCV MC-Vasc), 2.50 ± 0.60 (HCV), 3.90 ± 0.80 (HIV/HCV); *p* = 0.03 between HCV MC-Vasc and normal and *p* = 0.04 between HCV viremic and normal]. Defensins are proteins expressed in neutrophils that have broad anti-microbial properties (15). Our results suggest a selective depletion of the inflammatory capacity of neutrophils seen in patients with HCV infection. Although this down-regulation of alpha-defensin expression levels are not seen in the HIV/HCV coinfected cohort, alpha-defensins have been shown to be elevated in HIV-infected patients (16).

### HCV monoinfection up-regulates expression of genes involved in chemotaxis and response to external stimuli

**Figure 3:**
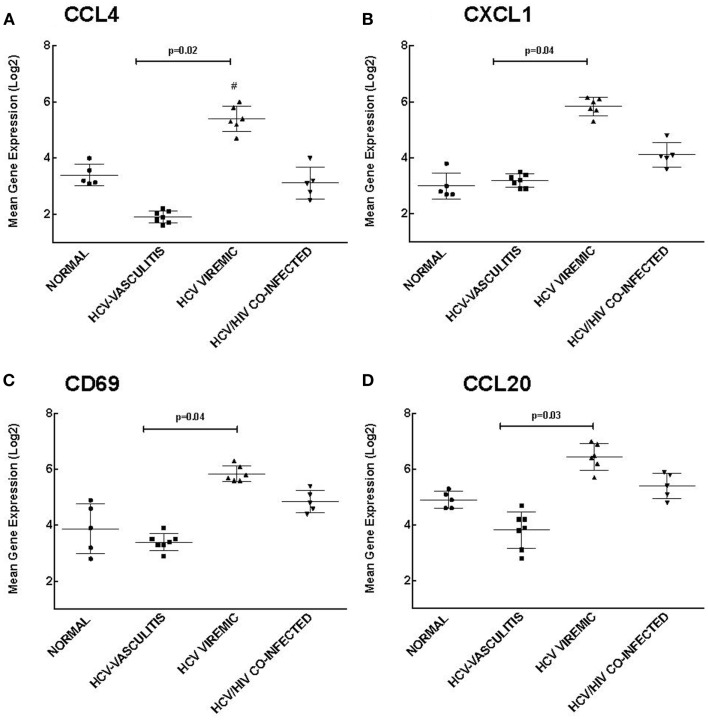
**Validation of Cluster 2 microarray data by qPCR analysis**. DNA microarray expression of biologically relevant genes selected from Cluster 2 was validated using qRT-PCR as described in the Section “Materials and Methods.” Levels of mean gene expression were calculated in comparison to that of *GAPDH*. The expression of CCL4 **(A)**, CXCL1 **(B)**, CD69 **(C)**, and CCL20 **(D)** were higher in the HCV monoinfected cohort compared to the other groups (*p* = 0.02, 0.04, 0.04, and 0.03, respectively compared to HCV MC-Vasc).

Cluster 2 is comprised of genes that are up-regulated in HCV monoinfected patients without vasculitis and play a role in chemotaxis and response to external stimuli. We validated the microarray analysis by comparing the expression levels of four genes in this cluster, chemokine (C-C motif) ligand 4 (CCL4), chemokine (C-X-C motif) ligand 1 (CXCL1), cluster of differentiation 69 (CD69), and chemokine (C-C motif) ligand 20 (CCL20) by qRT-PCR (Figure 3).

Chemokines are a family of cytokines responsible for mediating leukocyte chemotaxis (17). Transcript levels of CCL4, a lymphocyte attractant (18), were significantly higher in HCV viremic patients when compared to the three other subject cohorts [3.20 ± 0.80 (NV), 1.90 ± 0.70 (HCV MC-Vasc), 5.40 ± 0.90 (HCV), 2.80 ± 1.00 (HIV/HCV); *p* = 0.02 between HCV viremic and HCV MC-Vasc]. Similarly, CXCL1, a chemokine with neutrophil chemotactic activity (19), was up-regulated in HCV monoinfected patients [2.80 ± 0.90 (NV), 3.20 ± 0.60 (HCV MC-Vasc), 5.70 ± 0.50 (HCV), 4.10 ± 0.70 (HIV/HCV); *p* = 0.04 between HCV viremic and HCV MC-Vasc]. CD69 is a receptor that is induced upon antigen-associated activation of T cells and transmits signals to other lymphocytes (20). PBMC expression of CD69 was greater in the HCV monoinfected cohort [3.90 ± 0.80 (NV), 3.40 ± 0.90 (HCV MC-Vasc), 5.80 ± 0.80 (HCV), 4.80 ± 0.70 (HIV/HCV); *p* = 0.04 between HCV viremic and HCVMC-Vasc]. Finally, CCL20 showed similar gene expression patterns among the patient cohorts [4.90 ± 0.80 (NV), 3.80 ± 0.70 (HCV MC-Vasc), 6.50 ± 0.70 (HCV), 5.40 ± 0.80 (HIV/HCV); *p* = 0.03 between HCV viremic and HCV MC-Vasc]. These data further validate the microarray analysis and demonstrate that genes involved in chemotaxis and response to external stimuli are up-regulated in HCV monoinfected individuals relative to HCV MC vasculitis patients. In extension, these data suggest that the HCV MC vasculitis patient cohort has an impaired chemotactic response to stimuli.

### PBMCs of HCV-associated MC vasculitis patients show increased expression of ISGs

**Figure 4:**
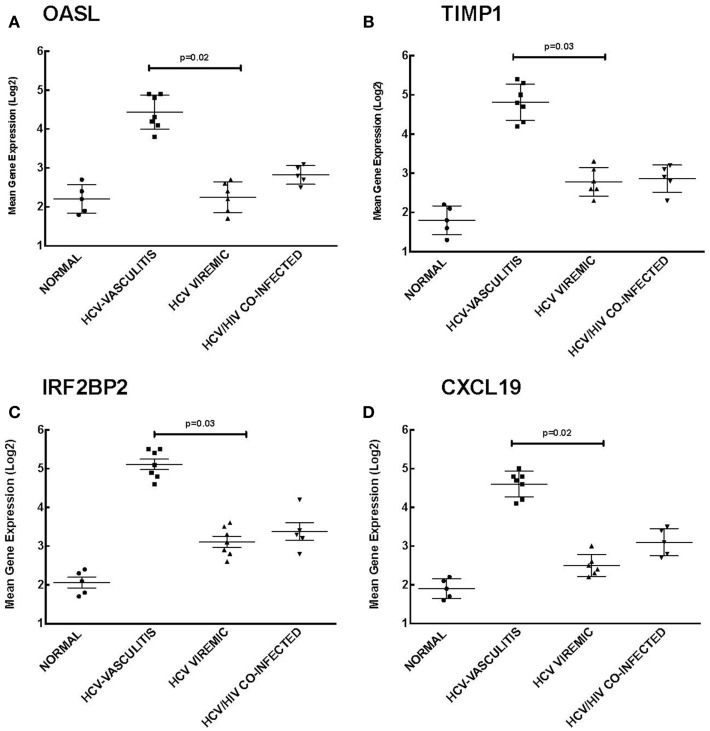
**Validation of Cluster 3 microarray data by qPCR analysis**. DNA microarray expression of four relevant ISGs selected from Cluster 3 was validated by qRT-PCR as described in the Section “Materials and Methods.” Levels of mean gene expression were calculated in comparison to that of *GAPDH*. The expression of OASL **(A)**, TIMP1 **(B)**, IRF2BP2 **(C)**, and CXCL16 **(D)** was up-regulated in the HCV MC vasculitis cohort compared to the other groups (*p* = 0.02, 0.03, 0.03, and 0.02, respectively between HCV MC-Vasc and HCV viremic).

Our analysis of Cluster 3 demonstrates an enrichment of ISGs in the HCV MC vasculitis cohort. We chose to validate the gene expression of 2′-5′-oligoadenylate synthetase-like protein (OASL), TIMP metallopeptidase inhibitor 1 (TIMP1), interferon regulatory factor 2 binding protein 2 (IRF2B2), and chemokine (C-X-C motif) ligand 16 (CXCL16) (Figure 4). OASL is an IFN-induced cellular protein that inhibits viral replication of HCV (21). Gene expression of OASL was higher in HCV MC vasculitis patients compared to the other subject cohorts [2.20 ± 0.50 (NV), 4.30 ± 0.90 (HCV MC-Vasc), 2.20 ± 0.80 (HCV), 2.70 ± 0.40 (HIV/HCV); *p* = 0.02 between HCV MC-Vasc and HCV viremic]. TIMP1 regulates extracellular matrix (ECM) turnover and remodeling by inhibiting the degradation of ECM (22). Transcript levels of TIMP1 were significantly higher in the HCV MC vasculitis cohort [1.80 ± 0.70 (NV), 4.8 ± 1.10 (HCV MC-Vasc), 2.80 ± 0.70 (HCV), 2.90 ± 1.00 (HIV/HCV); *p* = 0.03 between HCV MC-Vasc and HCV viremic]. Interferon regulatory factor 2 binding protein (IRF2BP2) is a co-repressor of type I IFN genes and many ISGs (23). The HCV MC vasculitis cohort had greater gene expression of IRF2BP2 when compared to the other study groups [2.10 ± 0.90 (NV), 5.10 ± 0.80 (HCV MC-Vasc), 3.10 ± 0.90 (HCV), 3.40 ± 0.80 (HIV/HCV); *p* = 0.03 between HCV MC-Vasc and HCV viremic]. Finally, CXCL16 is an interferon-gamma-regulated cytokine that promotes cell growth and migration and is also involved in lymphocyte chemotaxis (24). qRT-PCR validated the microarray analysis indicating that CXCL16 gene expression is up-regulated in HCV MC vasculitis patients [1.90 ± 0.60 (NV), 4.60 ± 0.80 (HCV MC-Vasc), 2.50 ± 0.60 (HCV), 3.10 ± 0.50 (HIV/HCV); *p* = 0.02 between HCV MC-Vasc and HCV viremic]. Thus, our data suggest that the MC vasculitis patient cohort express higher levels of ISGs at the transcript level than the other groups with or without HCV infection.

### Evidence for activation of monocytes in HCV-associated MC vasculitis

In order to determine which cells contribute to this increased ISG expression observed in the MC vasculitis patients, we performed enrichment and depletion studies in PBMCs for B cells and monocytes. The expression of ISGs in each cell subset was then quantified.

**Figure 5:**
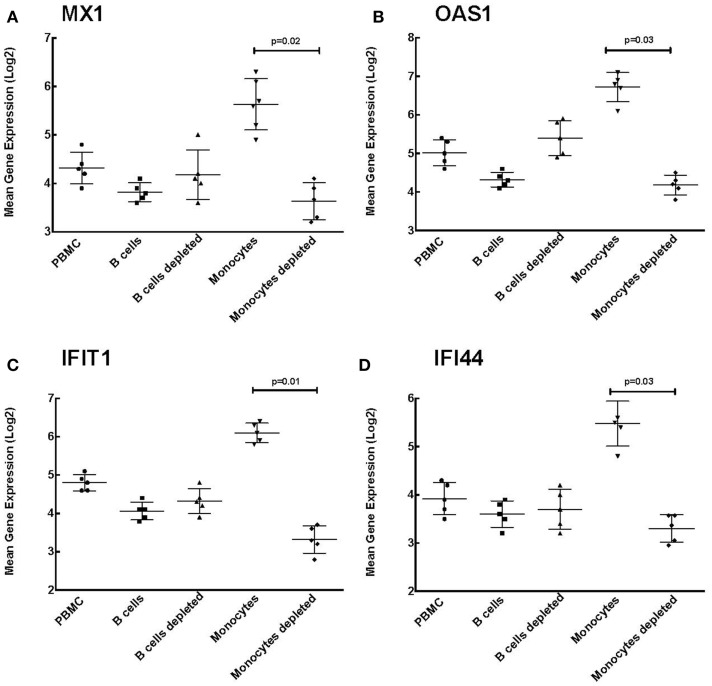
**Monocyte enrichment of PBMCs leads to increased ISG expression**. Expression of ISGs was quantified in total PBMCs, B cell enriched PBMCs, B cell depleted PBMCs, monocyte enriched PBMCs, and monocyte depleted PBMCs. Levels of mean gene expression of four selected ISGs, MX1 **(A)**, OAS1 **(B)**, IFIT1 **(C)**, and IFI44 **(D)**, were calculated in comparison to that of *GAPDH*. The expression of all four ISGs, MX1 (*p* = 0.02 between monocyte depleted and enriched), OAS1 (*p* = 0.03 between monocyte depleted and enriched), IFIT1 (*p* = 0.01 between monocyte depleted and enriched), and IFI44 (*p* = 0.03 between monocyte depleted and enriched), was significantly higher in monocyte enriched PBMCs compared to total PBMCs and monocyte depleted PBMCs, while no such differences were observed when B cells were depleted.

Our data indicate that B cell enrichment and depletion of PBMCs have no effect on ISG expression levels (Figure 5). As shown in Figure 5, the mean gene expression of ISGs MAX interactor 1 (MX1), 2′-5′-oligoadenylate synthetase (OAS1), interferon-induced protein with tetratricopeptide repeats 1 (IFIT1), and interferon-induced protein 44 (IFI44) was not statistically significant between PBMCs, B cell enriched PBMCs, and B cell depleted PBMCs. These results suggest a novel observation that the increase in ISG expression seen among the HCV MC vasculitis patient cohort is not directly due to the monoclonal expansion of B cells in MC vasculitis pathogenesis.

However, levels of MX1 expression were significantly higher in monocyte enriched PBMCs compared to the other cell subsets [4.30 ± 0.80 (PBMC), 3.80 ± 0.70 (B cells), 4.20 ± 1.00 (B cells depleted), 5.60 ± 0.80 (monocytes), and 3.67 ± 0.70 (monocytes depleted); *p* = 0.02 between monocyte enriched and depleted]. This was also true of OAS1 [5.00 ± 0.90 (PBMC), 4.30 ± 0.80 (B cells), 5.40 ± 0.90 (B cells depleted), 6.80 ± 0.60 (monocytes), and 4.20 ± 0.70 (monocytes depleted); *p* = 0.03 between monocyte enriched and depleted], IFIT1 [4.80 ± 0.60 (PBMC), 4.10 ± 0.60 (B cells), 4.30 ± 0.60 (B cells depleted), 6.10 ± 0.80 (monocytes), and 3.30 ± 0.60 (monocytes depleted); *p* = 0.01 between monocyte enriched and depleted], and IFI44 [3.90 ± 0.60 (PBMC), 3.60 ± 0.70 (B cells), 3.70 ± 0.50 (B cells depleted), 5.50 ± 0.40 (monocytes), and 3.30 ± 0.50 (monocytes depleted); *p* = 0.03 between monocyte enriched and depleted].

The higher expression levels of ISGs in monocyte enriched PBMCs compared to that seen in total PBMCs suggest that monocytes are likely responsible for the increased ISG expression among the HCV MC vasculitis cohort. Therefore, the probable mechanism underlying increased ISG expression is the indirect activation of monocytes by the monoclonal expansion of B cells in MC vasculitis.

## Discussion

Mixed cryoglobulinemic vasculitis is a common extrahepatic manifestation of CHC and can cause fulminant complications such as glomerulonephritis. CHC-associated MC vasculitis is characterized by HCV-driven monoclonal expansion of B cells producing IgM with rheumatoid factor activity (6, 9). These autoantibodies form complexes with circulating IgG and HCV particles, which are deposited in blood vessel and glomerular capillaries. The deposited cryoglobulins can stimulate the complement system and activate tissue damage and eventual end-organ damage, particularly of skin and kidneys (6, 9). However, not much is known about the mechanisms that trigger HCV induction of MC in some patients with CHC. Investigating the gene expression profile of HCV MC vasculitis patients is necessary to better elucidate the pathophysiology of CHC-induced MC vasculitis. In this study, we demonstrate that PBMCs from patients with HCV-associated MC vasculitis have a distinct gene expression profile when compared to PBMCs from patients with HCV and no vasculitis.

Overall, the gene expression profile of HCV-associated MC vasculitis reflects compromised neutrophil function, impaired chemotaxis, and an enhanced endogenous interferon gene signature. Notably, the peripheral ISG signature appears to be predominantly a result of the activation of monocytes, a novel observation that helps to elucidate the pathogenesis in HCV MC vasculitis patients. HCV MC vasculitis is thought to be primarily mediated by monoclonal expansion of B cells. In this study, we were able to demonstrate that other immune cells are also affected and contribute significantly to the disease pathogenesis. We have shown that elevated ISG expression, considered to be a bad prognostic factor for clearing HCV, is mediated by monocytes in HCV MC patients.

First, our microarray analysis suggests decreased cellular defense gene expression in HCV monoinfected subjects, with or without MC vasculitis. HCV has been shown to evade host immune mechanisms (25, 26), consistent with the down-regulation of defensins in the two HCV monoinfected patient groups. The enhanced antibody-dependent cell cytotoxicity associated with HCV MC vasculitis and leading to end-organ damage is mediated in part by neutrophils (27). These results suggest a depletion of alpha-defensin production in neutrophils either as a result of exhaustion or due to the influence of systemic corticosteroid administration, which some subjects were receiving at the time of the study. Due to the rare occurrence of HCV MC vasculitis and the lack of access to these patients, it was difficult to find a subject cohort who was not taking systemic corticosteroids, a limitation of our study. However, to ensure that the effects of systemic corticosteroids were minimal, we drew trough research samples 1 h before the daily dose of prednisone. Additionally, the clinical manifestations of HCV MC vasculitis were similar among all the patients whether or not they were taking corticosteroids.

Second, the presence of MC vasculitis appears to impair chemotaxis and response to external stimuli. The expression of several chemokines was down-regulated in HCV MC vasculitis patients when compared to HCV monoinfected patients. In particular, CCL20, involved in migration of lymphocytes to the liver (28), had a greater than twofold down-regulation at the transcript level in HCV MC vasculitis patients. These results suggest the presence of aberrant chemotactic pathways that exist in HCV-associated MC vasculitis as a result of an ongoing extrahepatic inflammatory response. In HCV-infected subjects, the liver is the primary organ of inflammatory response and the site of migration for immune cells. However, in patients with MC vasculitis and HCV infection, immune cells may in fact migrate to peripheral extrahepatic sites such as the kidneys and/or skin. Markers such as CD69 that represent early activation of lymphocytes are not up-regulated in the HCV MC vasculitis group, suggesting a distinct pattern of immune activation in HCV MC patients than that observed with HIV/HCV coinfected subjects.

Third, HCV MC vasculitis subjects also had increased expression of ISGs, an indicator of poor response to interferon-alpha-based therapy. B cell enrichment had no effect on ISG expression while monocyte enrichment of PBMCs led to significantly higher levels of ISG expression. This is a novel observation demonstrating that activation of monocytes may contribute to the pathogenic mechanisms in HCV MC vasculitis. Since most HCV MC vasculitis patients have undergone HCV treatment with interferon and ribavirin, it is probable that the enhanced ISG induction is an indicator of non-response and not specific to underlying vasculitis (29, 30). Hence, further analysis of PBMC gene expression profiles before and after *in vivo* B cell depletion is necessary to completely elucidate this pathway of monocyte activation and the role played by monoclonal B cells.

In summary, our study offers a preliminary analysis of the differential regulation of host gene expression in subjects with HCV-related MC vasculitis. Future studies will be focused on identifying the specific mechanisms behind the viral induction of B cell proliferation and the role of B cells in increased ISG expression in the context of B cell depletion therapy.

## Conflict of Interest Statement

The authors declare that the research was conducted in the absence of any commercial or financial relationships that could be construed as a potential conflict of interest.

## Appendix

**Table A1:** **Genes down regulated in all HCV subjects compared to normal**.

Gene ID	Name	Short name	Function
1554479_a_at	Caspase recruitment domain family, member 8	CARD8	Inhibits NF-kappa B activation. Suppression of apoptotic processes and inflammatory signaling pathways
203373_at	Suppressor of cytokine signaling 2	SOCS2	Negative regulator of apoptotic processes and cytokine signal transduction pathway
205798_at	Interleukin 7 receptor	IL7R	Involved in B cell and T cell proliferation and T cell development in thymus
205898_at	Chemokine (C-X3-C motif) receptor 1	CX3CR1	Mediates adhesion and migration of leukocytes such as monocytes, NK cells, and T lymphocytes
206978_at	Chemokine (C-C motif) receptor 2	CCR2	Monocyte chemotaxis
207269_at	Defensin, alpha 4, corticostatin	DEFA4	Expressed in the granule of neutrophils and has chemotactic and antimicrobial properties
217911_s_at	BCL2-associated athanogene 3	BAG3	Hsp70 co-chaperone and negative regulator of apoptosis. Also regulates development, cell motility, and autophagy
222201_s_at	CASP8 associated protein 2	CASP8AP2	Regulates apoptosis and cell cycle/survival
209728_at	Major histocompatibility complex, class II, DR beta 4	HLA-DRB4	Cell surface receptor that presents exogenous peptide antigens and is involved in T cell receptor signaling pathway
202018_s_at	Lactotransferrin /// similar to lactotransferrin	LOC728320 /// LTF	Glycoprotein that has antimicrobial and antiviral properties
205033_s_at	Defensin, alpha 1 /// defensin, alpha 3, neutrophil-specific /// defensin alpha 1	DEFA1 /// DEFA3 /// LOC653600 /// LOC728358	Expressed in the granule of neutrophils and has chemotactic and antimicrobial properties

**Table A2:** **Genes upregulated in HCV monoinfected subjects**.

Gene ID	Name	Short name	Function
202859_x_at	Interleukin 8	IL8	Chemotactic factor that attracts neutrophils, basophils, and T-cells and is involved in neutrophil activation
204103_at	Chemokine (C-C motif) ligand 4	CCL4	Monokine involved in cellular adhesion, signaling, and chemotaxis. Attracts NK cells and monocytes
204440_at	CD83 molecule	CD83	Surface marker for fully mature DC. Important for CD4(+) T cell development in the thymus
204470_at	Chemokine (C-X-C motif) ligand 1 (melanoma growth stimulating activity, alpha)	CXCL1	Chemotactic activity for neutrophils
205067_at	Interleukin 1, beta	IL1B	Produced by activated macrophages. Stimulates B-cell maturation and proliferation
205114_s_at	Chemokine (C-C motif) ligand 3 /// chemokine (C-C motif) ligand 3-like 1 /// che	CCL3 /// CCL3L1 /// CCL3L3 /// LOC728830 /// LOC730422	Monokine with inflammatory and chemokinetic properties
205207_at	Interleukin 6 (interferon, beta 2)	IL6	Cytokine that functions in inflammation and maturation of B lymphocytes
205476_at	Chemokine (C-C motif) ligand 20	CCL20	Chemotactic factor that attracts lymphocytes
207850_at	Chemokine (C-X-C motif) ligand 3	CXCL3	Chemotactic activity for neutrophils
209774_x_at	Chemokine (C-X-C motif) ligand 2	CXCL2	Chemokine produced by activated monocytes and neutrophils and expressed at sites of inflammation
210118_s_at	Interleukin 1, alpha	IL1A	Produced by activated macrophages and stimulates B cell maturation and proliferation
212657_s_at	Interleukin 1 receptor antagonist	IL1RN	Inhibits the activity of IL1
39402_at	Interleukin 1, beta	IL1B	Produced by activated macrophages and stimulates B cell maturation and proliferation
201925_s_at	CD55 molecule, decay accelerating factor for complement (Cromer blood group)	CD55	Integral membrane protein of erythrocytes, involved in regulation of complement activation
202643_s_at	Tumor necrosis factor, alpha-induced protein 3	TNFAIP3	Involved in immune and inflammatory responses signaled by cytokines through inhibition of NF-kappaB pathway
206359_at	Suppressor of cytokine signaling 3	SOCS3	Negative regulator of apoptotic processes and the insulin receptor signaling pathway
207008_at	Interleukin 8 receptor, beta	IL8RB	Activation of neutrophils
207535_s_at	Nuclear factor of kappa light polypeptide gene enhancer in B-cells 2 (p49/p100)	NFKB2	Transcription factor involved in immune and inflammatory pathways, cellular proliferation, cellular differentiation, and apoptosis
209795_at	CD69 molecule	CD69	Involved in lymphocyte proliferation
210354_at	Interferon, gamma	IFNG	Antiviral activity and several immunoregulatory functions
214637_at	Oncostatin M	OSM	Cytokine that regulates growth and is involved in hepatocyte differentiation

**Table A3:** **Genes up regulated in all HCV vasculitis subjects compared to others**.

Gene ID	Name	Short name	Function
201666_at	TIMP metallopeptidase inhibitor 1	TIMP1	Inhibitor of metalloproteinases that degrade extracellular matrix
205321_at	Eukaryotic translation initiation factor 2, subunit 3 gamma, 52 kDa	EIF2S3	Regulates rate of protein translation
205660_at	2′-5′-Oligoadenylate synthetase-like protein	OASL	IFN-induced cellular protein that inhibits viral replication of HCV and EMCV
217523_at	CD44 molecule (Indian blood group)	CD44	Mediates cell-to-cell and cell-to-matrix interactions. Involved in cellular migration and lymphocyte activation
223454_at	Chemokine (C-X-C motif) ligand 16	CXCL16	Transmembrane chemokine that promotes cell growth and migration, and involved in chemotaxis of T and NKT cells
224572_s_at	Interferon regulatory factor 2 binding protein 2	IRF2BP2	IRF-2 dependent transcriptional co-repressor of type I IFN genes and many IFN-stimulated genes
1554999_at	RasGEF domain family, member 1B	RASGEF1B	Regulation of small GTPase mediated signal transduction

## References

[1] Hadigan C Kottilil S Hepatitis C virus infection and coinfection with human immunodeficiency virus: challenges and advancements in management. JAMA (2011) 306:294–301 10.1001/jama.2011.975 21771990

[2] Ashfaq UA Javed T Rehman S Nawaz Z Riazuddin S An overview of HCV molecular biology, replication and immune responses. Virol J (2011) 8:161 10.1186/1743-422X-8-161 21477382 PMC3086852

[3] National institutes of health consensus development conference statement: management of hepatitis C 2002 (June 10-12, 2002). Gastroenterology (2002) 123:2082–99 10.1053/gast.2002.1232082 12454863

[4] Jacobson IM Cacoub P Dal Maso L Harrison SA Younossi ZM Manifestations of chronic hepatitis C virus infection beyond the liver. Clin Gastroenterol Hepatol (2010) 8:1017–29 10.1016/j.cgh.2010.08.026 20870037

[5] Galossi A Guarisco R Bellis L Puoti C Extrahepatic manifestations of chronic HCV infection. J Gastrointestin Liver Dis (2007) 16:65–73 17410291

[6] Dore MP Fattovich G Sepulveda AR Realdi G Cryoglobulinemia related to hepatitis C virus infection. Dig Dis Sci (2007) 52:897–907 10.1007/s10620-006-9510-9 17380399

[7] Ferri C La Civita L Longombardo G Greco F Bombardieri S Hepatitis C virus and mixed cryoglobulinaemia. Eur J Clin Invest (1993) 23:399–405 10.1111/j.1365-2362.1993.tb00782.x 8397090

[8] Saadoun D Bieche I Maisonobe T Asselah T Laurendeau I Piette JC Involvement of chemokines and type 1 cytokines in the pathogenesis of hepatitis C virus-associated mixed cryoglobulinemia vasculitis neuropathy. Arthritis Rheum (2005) 52:2917–25 10.1002/art.21270 16142759

[9] Ferri C Mascia MT Cryoglobulinemic vasculitis. Curr Opin Rheumatol (2006) 18:54–63 16344620 10.1097/01.bor.0000198002.42826.c2

[10] Lamprecht P Gause A Gross WL Cryoglobulinemic vasculitis. Arthritis Rheum (1999) 42:2507–16 10.1002/1529-0131(199912)42:12<2507::AID-ANR2>3.0.CO;2-# 10615995

[11] Antonelli A Ferri C Galeazzi M Giannitti C Manno D Mieli-Vergani G HCV infection: pathogenesis, clinical manifestations and therapy. Clin Exp Rheumatol (2008) 26:S39–47 18570753

[12] Saadoun D Delluc A Piette JC Cacoub P Treatment of hepatitis C-associated mixed cryoglobulinemia vasculitis. Curr Opin Rheumatol (2008) 20:23–8 10.1097/BOR.0b013e3282f1330c 18281853

[13] Sneller MC Hu Z Langford CA A randomized controlled trial of rituximab following failure of antiviral therapy for hepatitis C virus-associated cryoglobulinemic vasculitis. Arthritis Rheum (2012) 64:835–42 10.1002/art.34322 22147444 PMC3243106

[14] Kottilil S Yan MY Reitano KN Zhang X Lempicki R Roby G Human immunodeficiency virus and hepatitis C infections induce distinct immunologic imprints in peripheral mononuclear cells. Hepatology (2009) 50:34–45 10.1002/hep.23055 19551908 PMC2736098

[15] Ganz T Defensins: antimicrobial peptides of innate immunity. Nat Rev Immunol (2003) 3:710–20 10.1038/nri1180 12949495

[16] Rodriguez-Garcia M Climent N Oliva H Casanova V Franco R Leon A Increased alpha-defensins 1-3 production by dendritic cells in HIV-infected individuals is associated with slower disease progression. PLoS One (2010) 5:e9436 10.1371/journal.pone.0009436 20195543 PMC2828484

[17] Zlotnik A Yoshie O Nomiyama H The chemokine and chemokine receptor superfamilies and their molecular evolution. Genome Biol (2006) 7:243 10.1186/gb-2006-7-12-243 17201934 PMC1794421

[18] Lee SC Brummet ME Shahabuddin S Woodworth TG Georas SN Leiferman KM Cutaneous injection of human subjects with macrophage inflammatory protein-1 alpha induces significant recruitment of neutrophils and monocytes. J Immunol (2000) 164:3392–401 10.4049/jimmunol.164.6.3392 10706735

[19] De Filippo K Dudeck A Hasenberg M Nye E van Rooijen N Hartmann K Mast cell and macrophage chemokines CXCL1/CXCL2 control the early stage of neutrophil recruitment during tissue inflammation. Blood (2013) 121:4930–7 10.1182/blood-2013-02-486217 23645836

[20] Shinoda K Tokoyoda K Hanazawa A Hayashizaki K Zehentmeier S Hosokawa H Type II membrane protein CD69 regulates the formation of resting T-helper memory. Proc Natl Acad Sci U S A (2012) 109:7409–14 10.1073/pnas.1118539109 22474373 PMC3358871

[21] Ishibashi M Wakita T Esumi M 2’,5’-Oligoadenylate synthetase-like gene highly induced by hepatitis C virus infection in human liver is inhibitory to viral replication *in vitro*. Biochem Biophys Res Commun (2010) 392:397–402 10.1016/j.bbrc.2010.01.034 20074559

[22] Neuman MG Schmilovitz-Weiss H Hilzenrat N Bourliere M Marcellin P Trepo C Markers of inflammation and fibrosis in alcoholic hepatitis and viral hepatitis C. Int J Hepatol (2012) 2012:231210 10.1155/2012/231210 22530132 PMC3296182

[23] Childs KS Goodbourn S Identification of novel co-repressor molecules for interferon regulatory factor-2. Nucleic Acids Res (2003) 31:3016–26 10.1093/nar/gkg431 12799427 PMC162335

[24] Matloubian M David A Engel S Ryan JE Cyster JG A transmembrane CXC chemokine is a ligand for HIV-coreceptor Bonzo. Nat Immunol (2000) 1:298–304 10.1038/79738 11017100

[25] Chen Y Chen J Wang H Shi J Wu K Liu S HCV-induced miR-21 contributes to evasion of host immune system by targeting MyD88 and IRAK1. PLoS Pathog (2013) 9:e1003248 10.1371/journal.ppat.1003248 23633945 PMC3635988

[26] Larrea E Riezu-Boj JI Aldabe R Guembe L Echeverria I Balasiddaiah A Dysregulation of interferon regulatory factors impairs the expression of immunostimulatory molecules in hepatitis C virus genotype 1-infected hepatocytes. Gut (2013) 63(4):665–73 10.1136/gutjnl-2012-304377 23787026

[27] Cacoub P Renou C Rosenthal E Cohen P Loury I Loustaud-Ratti V Extrahepatic manifestations associated with hepatitis C virus infection. A prospective multicenter study of 321 patients. The GERMIVIC. Groupe d’Etude et de Recherche en Medecine Interne et Maladies Infectieuses sur le Virus de l’Hepatite C. Medicine (Baltimore) (2000) 79:47–56 10.1097/00005792-200001000-00005 10670409

[28] Oo YH Banz V Kavanagh D Liaskou E Withers DR Humphreys E CXCR3-dependent recruitment and CCR6-mediated positioning of Th-17 cells in the inflamed liver. J Hepatol (2012) 57:1044–51 10.1016/j.jhep.2012.07.008 22796894 PMC3994510

[29] Chen L Borozan I Feld J Sun J Tannis LL Coltescu C Hepatic gene expression discriminates responders and nonresponders in treatment of chronic hepatitis C viral infection. Gastroenterology (2005) 128:1437–44 10.1053/j.gastro.2005.01.059 15887125

[30] Lempicki RA Polis MA Yang J McLaughlin M Koratich C Huang DW Gene expression profiles in hepatitis C virus (HCV) and HIV coinfection: class prediction analyses before treatment predict the outcome of anti-HCV therapy among HIV-coinfected persons. J Infect Dis (2006) 193:1172–7 10.1086/501365 16544259

